# Fatal toxoplasmic encephalitis triggered by anti-TNF therapy

**DOI:** 10.1016/j.heliyon.2025.e41965

**Published:** 2025-01-21

**Authors:** Rodrigo A. Montoro, Michael Moran, Katherine A. Overmyer, Andrew Periaccante, Joshua J. Coon, Swapnil Lanjewar, Laura J. Knoll, Rob Striker

**Affiliations:** a Department of Medical Microbiology and Immunology, University of Wisconsin-Madison, 1550 Linden Drive, Madison, WI, 53706, United States; b Department of Medicine, University of Wisconsin-Madison, United States; c Department of Biochemistry, University of Wisconsin-Madison, United States

**Keywords:** Anti-TNF-α therapy, *Toxoplasma*, Encephalitis, Proteomics

## Abstract

Reactivation of a latent infection by the protozoan parasite *Toxoplasma gondii* can result in severe neurologic outcomes and even death. *T. gondii* reactivation cases have been strongly associated with acquired immunodeficiency syndrome, but other immunosuppressive situations are also associated. Anti-TNF-α therapy reliably triggers the reactivation of *T. gondii* latent cysts in mice models. Reactivation of *T. gondii* by TNF-a blockade is rare in humans though despite widespread use of TNF-a blockers. Serologic evidence of a possible latent *T. gondii* infection in humans is common worldwide, so why anti-TNF-α reactivation isn't more common is unknown. Here we present a 74-year-old woman who developed fatal cerebral toxoplasmosis after anti-tumor necrosis factor-α (TNF-α). After presenting to a local urgent care with confusion, a worsening cognitive status led to an emergency room visit. Computed tomography resulted in suspicion of metastatic disease leading to treatment with steroids. Lumbar puncture ruled out bacterial or viral meningitis. With continued cognitive decline, magnetic resonance imaging of the head revealed an increased number of lesions with *T. gondii*-associated ring-enhancing lesions. A brain biopsy confirmed the presence of *T. gondii* parasites. Despite standard treatment for toxoplasmosis, the patient expired. At least two possible factors may have contributed to this unfortunate outcome. First, in addition to her rheumatoid arthritis pathology, there is evidence of loss of immune resilience and abnormal T cell subsets. Second, some strains of *T. gondii* are more virulent than others. Post-mortem mass spectrometry and proteomic analysis of her cerebrospinal fluid show several *T. gondii* peptides. A literature review suggests that risks associated with anti-TNF-α therapy for patients who are seropositive for *T. gondii* have not been adequately recognized. We advise *T. gondii* seropositivity testing be considered before and after initiation of anti-TNF-α therapy as is done for other infections such as tuberculosis.

## 1 Introduction

Anti-tumor necrosis factor alpha (TNF-ɑ) medications have provided significant relief from a variety of inflammatory disorders, and their use is now common [1]. However, the immunosuppressive effects of these drugs carry an increased risk of specific infections [2].

*Toxoplasma gondii* is a protozoan parasite found globally. Serologic studies indicate that approximately 30 % of the world's population may have a chronic *T. gondii* infection [3]. Chronic *T. gondii* infections are generally asymptomatic. *T. gondii* infection usually only reactivates and causes encephalitis in severely immunocompromised individuals, such as end-stage acquired immunodeficiency syndrome (AIDS) patients with an average CD4 count of <50 cells/mL. *T. gondii*-infected TNF-ɑ knockout mice have severe outcomes, and treatment of mice with chronic *T. gondii* infection with TNF-ɑ inhibitors causes reactivation and death [4,5]. While case reports of *T. gondii* reactivation after the use of anti-TNF-ɑ therapies have been documented [[6], [7], [8]], they are not common. Here we present a case of a 74-year-old patient with rheumatoid arthritis on intermittent anti-TNF-ɑ therapy who developed encephalitis from *T. gondii* and expired. We provide evidence that proteomic analysis of cerebrospinal fluid could potentially be used in the future to help risk stratify the large quantity of *T. gondii* seropositive patients on anti-TNF-ɑ therapy or in other diseases with hypothesized links to *T. gondii*, such as schizophrenia [9].

## 2 Case presentation

A 74-year-old female, Mexican-born, was diagnosed (1978 in Mexico) with rheumatoid arthritis and received methotrexate. She first received an anti-TNF-α therapy in 2015 and was intermittently on either infliximab (300 mg) or certolizumab (400 mg) until 2017. In 2021 she was restarted on infliximab and in July 2023 she presented to an emergency department for progressive altered mentation.

**Fig. 1:**
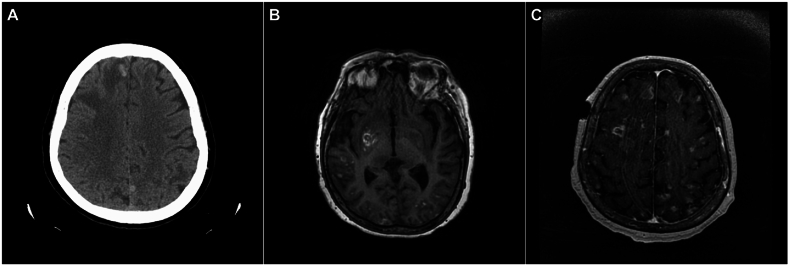
Computed tomography and magnetic resonance imaging of patient with reactivated cerebral toxoplasmosis. Initial CT neuroimaging of patient showed scattered intracranial lesions (1A). MRI found ring enhancement surrounding lesions with similar hemorrhaging pattern (T1-weighted) (1B). Follow-up MRI imaging 1-week post treatment initiation showed innumerable lesions (T1-weighted) (1C).

Prior to her emergency room presentation, she reported to a local urgent care for mild confusion and was treated for an uncomplicated urinary tract infection empirically. As her confusion progressed, she reported to the local emergency department mentioned above where computed tomography (CT) imaging of the head showed multiple scattered intracranial lesions with surrounding edema concerning hemorrhagic metastatic malignant lesions (Fig. 1A). She was admitted with plans for an open biopsy of the lesions, and steroids (dexamethasone 2–10 mg) were started. Lumbar puncture showed a pleocytosis (90 nucleated cells/μL) with 99 % lymphocytes, an opening pressure of 17, and a negative PCR analysis for common bacterial and viral causes of meningitis. Routine labs revealed only a mild thrombocytopenia (148,000/μL) with no leukocytosis (5100/μL). On bedside examination the patient was disoriented, minimally conversant, and did not follow verbal commands. She had no localizing strength defects. On discussion with the family, she had no recent travel or livestock exposure, and was last in Mexico years prior. She did have cats when she lived in Mexico and had not eaten raw meat or unpasteurized dairy products.

A presumptive diagnosis of metastatic disease was made. The patient was transferred to our institution for further care. Before she could be biopsied, she developed acute left-sided hemiparesis and neglect with slurred speech. Follow-up magnetic resonance imaging (MRI) of the brain (15 days later) showed multiple ring-enhancing parenchymal lesions with edema and similar hemorrhage (Fig. 1B). The following day a biopsy of one lesion showed abundant lymphocytes, macrophages, and *T. gondii* in both the rapidly replicating tachyzoites and cyst forms on microscopy. Immediately after diagnosis of cerebral toxoplasmosis pyrimethamine (75–200 mg), sulfadiazine (1500 mg), and leucovorin (25–50 mg) were started orally. An immunodeficiency evaluation revealed a CD4/CD8 count of 386/888 with an abnormally low ratio of 0.3, indicating an immune health grade of IV [10]. Immunoglobulin titers were notable for an IgG of 336 (mg/dL) and was given a single infusion of IVIG. A serum HIV antigen/antibody test was non-reactive. Within days of initiating therapy her mental status declined further to a Glasgow Coma Score of 4. A repeat MRI of the brain was performed one week after starting therapy which showed a new right internal capsule infarct and innumerable enhancing lesions throughout the brain (Fig. 1C). As the patient continued to decline the family eventually opted for hospice as she became further obtunded and eventually expired.

## 3 Discussion

*T. gondii* typically propagates clonally. Analysis of *T. gondii* genetic variation has been dominated by the archetypal “Three clones (I,II, and III) hypothesis” [11]. More recent classification using additional genetic loci shows that recombinant nonarchetypal strains exist in both human cases and wild animals [12]. Early on in the AIDS epidemic, it appeared that type II strains represented most of the human infections in North America and Europe. Recently, type I strains or strains containing type I alleles have been detected in immunocompromised individuals [13]. With improving molecular diagnostic techniques, nonarchetypal strains of *T. gondii* have been detected within the US [14], with a surprisingly high rate. Genotyping tools have demonstrated these outbreaks to be caused by atypical, highly virulent strains, as defined in mouse bioassays. The diversification of *T. gondii* strains has been attributed to recombinant events occurring within the Amazonian basin [11]. Brazil. in particular, has repeatedly been afflicted by outbreaks of nonarchetypal strains of *T. gondii*. Still nonarchetypal strains have been found in distant locations, including sub-Saharan Africa, Turkey, and Pacific Rim countries [11,15].

The specific genotype of the *T. gondii* strain that resulted in the fatal outcome presented here remains unknown. It is relatively rare for patients on anti-TNF-α therapy to have *T. gondii* reactivation, which may be due to seropositive individuals having nonviable *T. gondii* cysts in their neural tissues. Given the extreme outcome seen in this patient, we hypothesize they may have been infected with a nonarchetypal strain, which was able to reactivate on anti-TNF-α therapy. Post-mortem mass spectroscopic proteomic analysis of the patient's cerebrospinal fluid (CSF) – using previously described methodologies [[16], [17], [18]]. For proteomics data processing, raw files were searched using the MaxQuant quantitative software package. Fourteen FASTA files downloaded from ToxoDB on September 22, 2023 were able to detect *T. gondii-*specific peptide sequences not readily aligned to the archetypal types I, II, or III strains. As shown in Table 1, peptide sequences discovered by mass spectroscopy and later analyzed for sequence alignment through BLASTp query identified five *T. gondii*-specific peptides belonging to various protein families. Among these evaluated peptides, three strains of *T. gondii* had significant alignment with all 5 peptides, the clinically relevant type II strain, ME49, a type I strain, GT1, and a presumably nonarchetypal strain, COUG.

**Table 1:** Summary table of characterized *Toxoplasma gondii* proteins detected on proteomic analysis of CSF and *T. gondii* strains identified through cross-referenced BLASTp analysis. Bolded in red are strains that appeared in all evaluated peptides significant in CSF proteomic and BLASTp analyses.

Peptide Sequence Identified from Proteomic Analysis	Associated Protein ID	Proteomic Analysis Confidence Score	Toxoplasma Strains Identified from BLASTp Cross-Reference Analysis	BLASTp E value	ToxoDB PCR-RFLP Genotype	ToxoDB Signal Peptide
LSASLQPATLSKSEDGYLHFGQR	EF hand family protein	∗∗∗	**COUG**	1.00E-15	N/A	No
			**GT1**		I	No
			MAS		I, II, III, u-1	No
			**ME49**		II	No
TGTAPEMYGTKTASRR	threonine synthase	∗∗∗	ARI	5.00E-09	I, II, u-1	No
			CAST		I, II, III	No
			**COUG**		N/A	No
			**GT1**		I	No
			**ME49**		II	No
			p89		N/A	No
			RH		I	No
			RUB		I, II, III	No
			VEG		II, III	No
EVSDLVAELERK	hypothetical protein	∗∗∗∗	ARI	1.00E-04	I, II, u-1	No
			**COUG**		N/A	No
			GAB2-2007-GAL-DOM2		N/A	No
			**GT1**		I	No
			**ME49**		II	No
			p89		N/A	No
			TgCatBr9		N/A	No
			TgCatPRC2		N/A	No
	putative trichohyalin		VEG		II, III	No
VSDSATSVSISSSQAILSR	diacylglycerol acyltransferase	∗∗∗	ARI	3.00E-10	I, II, u-1	No
			**COUG**		N/A	No
			**GT1**		I	No
			**ME49**		II	No
			TgCatPRC2		N/A	No
			VAND		I, II, III	No
			CAST	4.00E-09	I, II, III	No
SLCSLRSPESENEVTGK	DIE2/alg10 family protein	∗∗∗∗	RUB	1.00E-09	I, II, III	No
			ARI	9.00E-08	I, II, u-1	No
			**COUG**		N/A	No
			FOU		I, II, III, u-1	No
			**GT1**		I	No
			MAS		I, II, III, u-1	No
			TgCatPRC2		N/A	**Yes**
			VAND		I, II, III	No
			VEG		II, III	No
	hypothetical protein		**ME49**		II	No

To our knowledge, this is the first demonstration of *T. gondii* peptides in human CSF. Further research is needed to determine if this modality of molecular analysis, without a brain biopsy, has high sensitivity as false negative PCR for *T. gondii* have been reported [19]. This case should raise concern for the potential of *T. gondii* strains with increased virulence and more adverse outcomes.

While different *T. gondii* strains exist both in wild and domesticated animals, slow progress has demonstrated certain haplotypes are particularly clinically important [11,12]. Specifically, haplotype 12 (a nonarchetypal strain) appears to be particularly virulent thus, research using serology or other methodologies to identify virulent haplotypes from less virulent strains is needed.

With the rising concern of nonarchetypal strains of *T. gondii*, at-risk populations such as older individuals and immunocompromised patients need updated guidelines for infection prevention when using anti-TNF-α therapy. Given the potential for reactivation of chronic *T. gondii* infections by anti-TNF-ɑ therapy; we cataloged several encephalitic patients similar to the one presented here, and documented elsewhere [6,7,[20], [21], [22], [23]] (Supplementary Table 1). There is also potential for less severe retinal presentations [8]. Patients who are serologically positive for *T. gondii* should be counseled on the risk that anti-TNF-α therapy can pose to their health, similar to how patients with tuberculosis are advised. A consensus has been reached that individuals starting anti-TNF-α therapy should be screened for latent tuberculosis before therapy initiation [24]. Due to the potential risk that anti-TNF-α therapy may pose, we suggest additional screening for *T. gondii* and recommend *T. gondii* seropositive patients receive regular reassessment of anti-TNF-α therapy or should be considered for secondary prophylaxis with trimethoprim/sulfamethoxazole to prevent these outcomes.

## 4 Conclusion

This case demonstrates the potential risk that immune suppression has for specific infections. Patients with latent *T. gondii* infections may be at greater risk for reactivation of toxoplasmosis if they are on anti-TNF-α therapy. As is currently done in the care of patients with tuberculosis, we suggest increased considerations and caution be taken when prescribing or maintaining *T. gondii* seropositive patients on anti-TNF-α therapy.

## CRediT authorship contribution statement

**Rodrigo A. Montoro:** Writing – review & editing, Writing – original draft, Data curation. **Michael Moran:** Writing – review & editing, Writing – original draft, Investigation. **Katherine A. Overmyer:** Writing – review & editing, Methodology, Investigation. **Andrew Periaccante:** Methodology, Investigation. **Joshua J. Coon:** Writing – review & editing, Methodology. **Swapnil Lanjewar:** Investigation. **Laura J. Knoll:** Writing – review & editing, Conceptualization. **Rob Striker:** Writing – review & editing, Conceptualization.

## Declaration of competing interest

The authors declare the following financial interests/personal relationships which may be considered as potential competing interests:Dr. Striker is a consultant for Pfizer, Salus Discovery and Voiant.
