# Supplementary material for: Fatal toxoplasmic encephalitis triggered by anti-TNF therapy
Source: Heliyon. 2025 Jan 21;11(3):e41965. doi: 10.1016/j.heliyon.2025.e41965 (PMC11830312; doi:10.1016/j.heliyon.2025.e41965)

**Supplemental Data:**

**Supplementary Table 1:** Case reports of reactivated cerebral toxoplasmosis after TNF blockade.


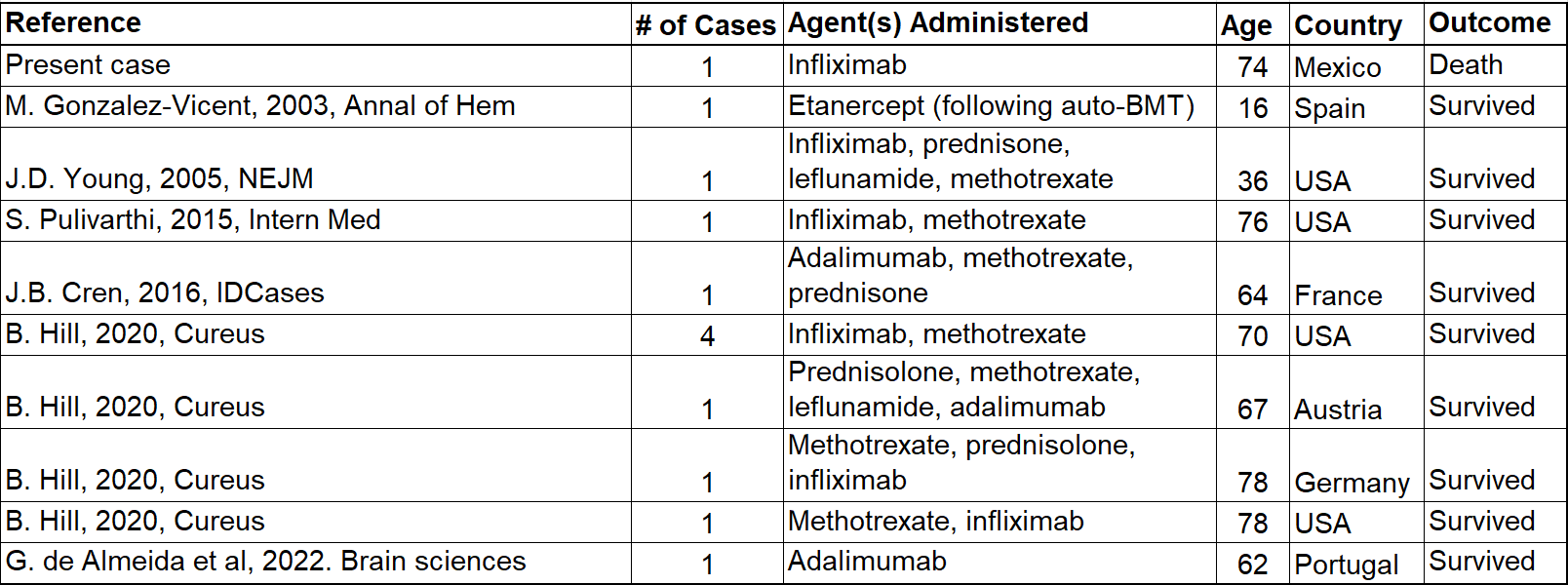

Supplement: Multimedia component 1 [file mmc1.docx]
